# Supplementary figures and images for: Development of an antigen detection assay for early point-of-care diagnosis of Zaire ebolavirus
Source: PLoS Negl Trop Dis. 2020 Nov 3;14(11):e0008817. doi: 10.1371/journal.pntd.0008817 (PMC7608863; doi:10.1371/journal.pntd.0008817)

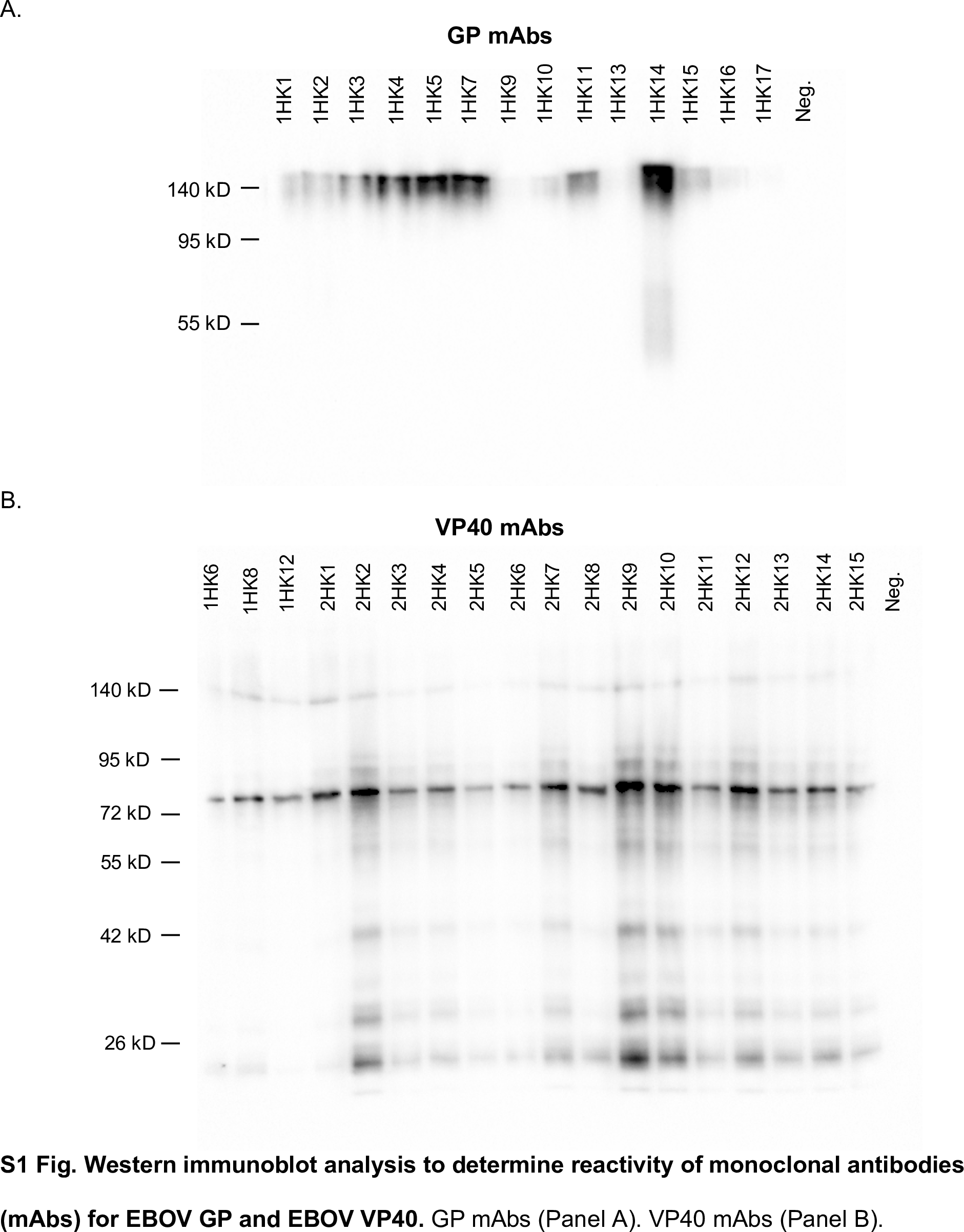

Supplement: S1 Fig — GP mAbs (Panel A) and VP40 mAbs (Panel B) were assessed for reactivity using Ebola virus-like particles (1 ug). (TIFF) [file pntd.0008817.s001.tiff]

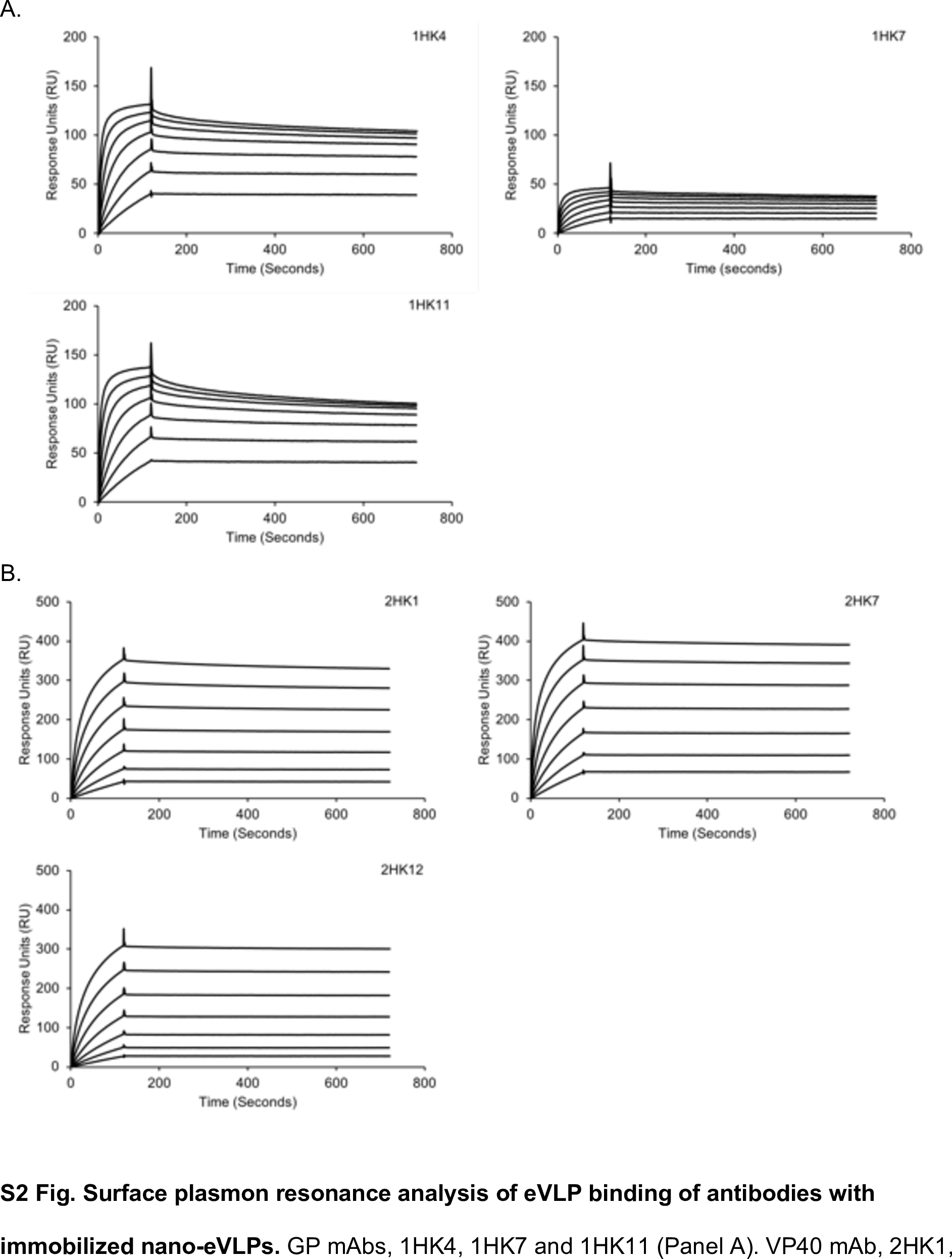

Supplement: S2 Fig — Panel A–GP mAbs: 1HK4, 1HK7 and 1HK11. Panel B–VP40 mAbs: 2HK1, 2HK7 and 2HK12. Data shown is from a single experiment representative of three independent experiments. (TIFF) [file pntd.0008817.s002.tiff]

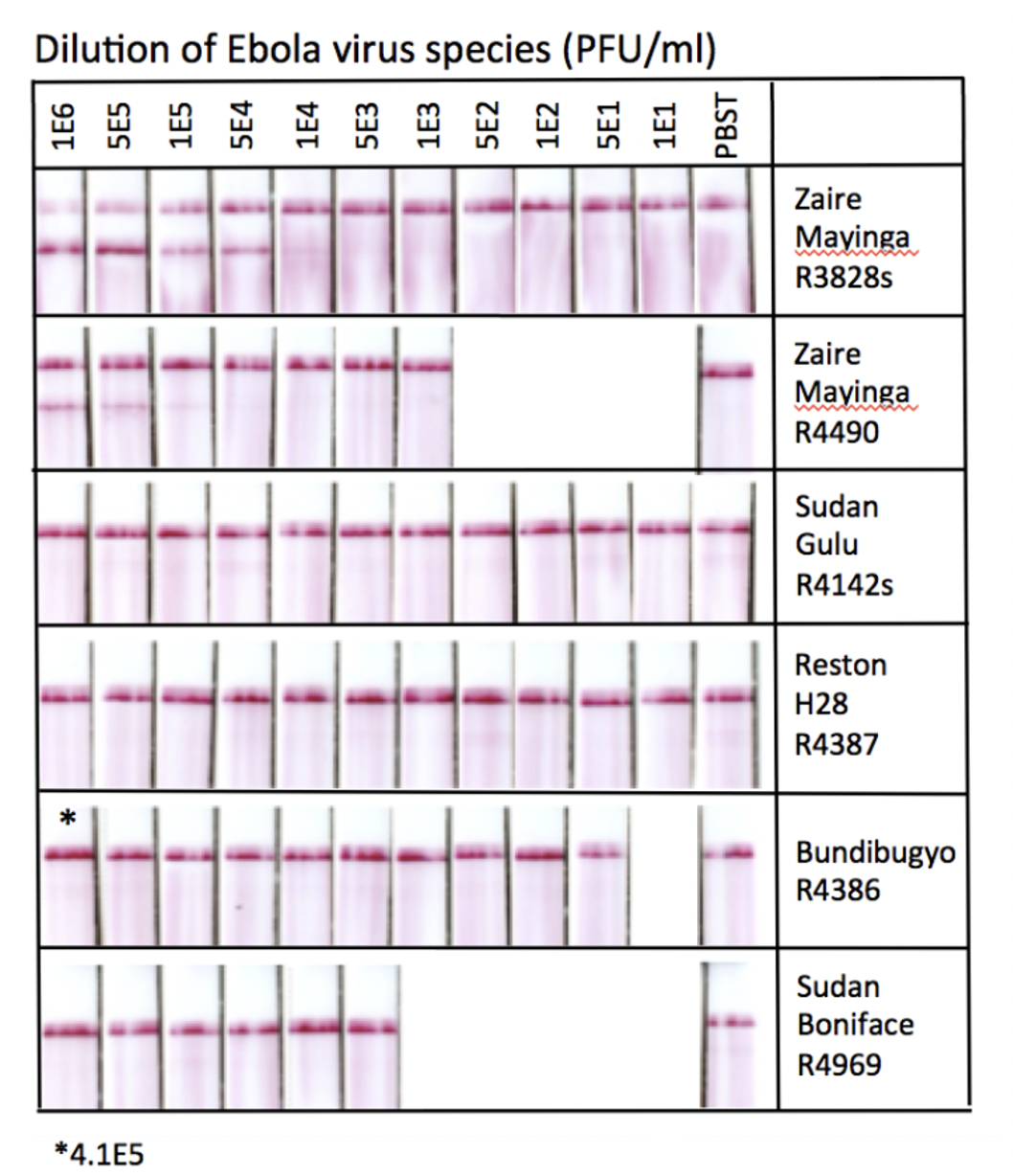

Supplement: S3 Fig — Dilutions of inactivated Ebola virus species (e.g. 1E6 = 1 x 10^6^ PFU/ml) were tested on a GP LFI prototype. This prototype was developed using 1HK7 (capture):1HK11 (detection) GP mAbs. The sample did not run optimally, note the streaking of gold labeled 1HK11 below the control (highest reactive line on each LFI). Empty areas within the figure indicate that these dilutions were not analyzed. (TIFF) [file pntd.0008817.s003.tiff]
