# Supplementary material for: Development of an antigen detection assay for early point-of-care diagnosis of Zaire ebolavirus
Source: PLoS Negl Trop Dis. 2020 Nov 3;14(11):e0008817. doi: 10.1371/journal.pntd.0008817 (PMC7608863; doi:10.1371/journal.pntd.0008817)
Supplement: S1 Table — (DOCX) [file pntd.0008817.s004.docx]

**S1 Table. Preliminary EBOV antigen-capture ELISAs evaluating sensitivity of GP and VP40 mAb pairs with virus-like particles (ng/ml).**

| **GP mAbs** | | | | | | | |
| --- | --- | --- | --- | --- | --- | --- | --- |
|  |  | Capture mAb | | | | | |
| Detection HRP mAb |  | 1HK1 | 1HK3 | 1HK4 | 1HK5 | 1HK7 | 1HK11 |
|  | 1HK1 | >1000 | 51 | 150 | >1000 | 83 | 51 |
|  | 1HK3 | 84 | 71 | 140 | >1000 | 30 | 76 |
|  | 1HK4 | 64 | 34 | 110 | >1000 | 23 | 80 |
|  | 1HK5 | 45 | 78 | 96 | >1000 | 21 | 41 |
|  | 1HK7 | 170 | 66 | 92 | >1000 | 290 | 55 |
|  | 1HK11 | 120 | 77 | 10 | >1000 | 19 | 35 |

| **VP40 mAbs** | | | | | | | |
| --- | --- | --- | --- | --- | --- | --- | --- |
|  |  | Capture mAb | | | | | |
| Detection HRP mAb |  | 1HK8 | 1HK12 | 2HK1 | 2HK2 | 2HK7 | 2HK12 |
|  | 1HK8 | 40 | 80 | 60 | 37 | 51 | 44 |
|  | 1HK12 | 64 | 63 | 98 | 56 | 84 | 63 |
|  | 2HK1 | 55 | 38 | 160 | 27 | 28 | 19 |
|  | 2HK2 | 49 | 64 | 43 | 39 | 40 | 36 |
|  | 2HK7 | 59 | 61 | 21 | 47 | 65 | 42 |
|  | 2HK12 | 33 | 39 | 57 | 22 | 42 | 38 |
