# Supplementary material for: Development of an antigen detection assay for early point-of-care diagnosis of Zaire ebolavirus
Source: PLoS Negl Trop Dis. 2020 Nov 3;14(11):e0008817. doi: 10.1371/journal.pntd.0008817 (PMC7608863; doi:10.1371/journal.pntd.0008817)
Supplement: Attachment — Submitted filename: letter.pdf [file pntd.0008817.s005.pdf]

University of Nevada, Reno
School of Medicine

Masayuki Saijo MD, PhD
Samuel Scarpino, PhD
Editors - PLoS NTD

August 17, 2020

Dear Drs. Siajo and Scarpino,

Thank you for editing our manuscript "Development of an antigen detection assay for early point-of-care diagnosis of *Zaire ebolavirus*". In addition, please thank the reviewers for the thoughtful and helpful critiques. Please forgive the delay in returning this revision. Similar to all other researchers, we are struggling to return to normal research activities during the COVID-19 pandemic.

We would like to offer a revised manuscript for your consideration. There have been major changes to the manuscript and most of the recommendations by the reviewers have been addressed. Most notably, we have included extra data on reactivity with other Ebola virus species via lateral flow immunoassay. This work was performed at USAMRIID and confirmed that the top LFI prototype is specific to Ebolavirus Zaire. We do hope that the updates to the manuscript will warrant publication.

This work will continue as it was just funded by an NIAID STTR small business award granted to InBios International and the University of Nevada, Reno. We hope to fully validate the performance of our top candidate LFIs over the next two years.

Below is a point-by-point response to the reviewer's comments.

Thank you for considering this revision,

Dave

David P. AuCoin, Ph.D.
Professor & Chair
Department of Microbiology and Immunology
University of Nevada School of Medicine

DeMers et al. Response to reviewers

Reviewer #1: Please see comments below.

Reviewer #2: There is a big difference in the band size of VP40 between Fig. 1 and S1 Fig. This should be explained.

Response: Good point, this should have been explained. In Figure 1 recombinant VP40 was probed by Western blot while in Supplemental Figure S1 purified virus like particles were used. We did find the difference in molecular weights interesting, however it is well established that VP40 can form dimers and oligomers. This was explained in the text and references were added following the text that described Supplemental Figure 1 (lines 324-328).

Lines 132-134: Variant name of the cloned genes (GP and VP40) should be provided.

Response: Zaire was added to the text for both cloned genes and the GenBank accession number was also added.

Reviewer #3: 1. It is not described how to pre-treat EBOV samples before loading onto LFI strip. The information will be important to understand the accessibility of monoclonal antibodies to epitopes of GP or VL40.

Response: EBOV samples were not pretreated prior to application on the LFI. Samples (live virus from cell culture or serum from infected NHPs) were thawed (added to text) and diluted in chase buffer if needed.

Results

Reviewer #1: Please see comments below.

Reviewer #2:

Major Comments:

1. The major issue with this study is the lack of confirmation for the specificity of the LFI test. Since the Makona variant is phylogenetically distinct from the other Ebola viruses in Zaire ebolavirus species, other variants (e.g., Mayinga, Kikwit, etc.) should also be tested for the sensitivity of the LFI assay. In addition, information on the reactivity of the antibodies to other ebolaviruses (e.g., Sudan and Bundibugyo viruses) should be provided.

Response: We agree that the specificity of the assay is a critical parameter to study. We added Western blot data testing all of the mAbs against Sudan Boniface, Täi Forest, Bundibuyo, Reston to Table 1. We also added GP LFI data that was generated a USAMRIID with different ebolavirus species (Mayinga, Sudan Gulu, Sudan Boniface, Reston, Bundibugyo. Although the protype LFIs did not run optimally they did show clear reactivity with Zaire species only. We included this as supplemental figure S3. We did not send VP40 LFIs to USAMRIID as the GP LFI prototype was most encouraging.

2. Considering the practical use, some other viral, bacterial, and protozoal pathogens that cause similar symptoms (other hemorrhagic fever viruses, Leptospira, Plasmodium, etc.) should also be tested to confirm the specificity of the LFI assay.

This is a good point. We did not test other microbes that cause similar symptoms to Ebola at this point. The antibodies have been shipped to InBios International where they will test the antibodies against a panel of microbes in order to complete a full specificity analysis. We have updated lines ~630-634 to reflect these plans.

Reviewer #3: 1. This study did not show epitopes of corresponding monoclonal antibodies. Therefore, the cross-reactivity to other ebolavirus species is unpredictable.

Response: Yes, this is true. We plan on mapping the epitopes of the mAbs that will be potentially be used in the final LFI. However, we were able to include new data on reactivity of the GP mAbs to other ebolavirus species.

2. The stability of monoclonal antibodies dried in LFI strip

was not described. Although the stability is generally recognized, the stability in terms of the reactivity of monoclonal antibodies might be varied among different monoclonal antibodies.

Response: This type of testing is usually addressed in the final stages of product development. We did however add to the introduction that LFIs usually are stable on the shelf for 18 months.

3. The manuscript mainly described for the development of LFI, while authors described about the antigen-capture ELISA system as well. Although the use of two monoclonal antibodies is overlapped, it is not clear why the demonstration of the antigen-capture ELISA system is required in this manuscript. Unless the sensitivity is compared between antigen-capture ELISA system and LFI, the antigen-capture ELISA description can be moved to supplemental data.

Response: We do like to compare the LOD of our antigen-capture ELISA to that of our LFI. In addition, we feel that there may be potential to develop an antigen-capture ELISA for the testing of hundreds of samples in endemic regions if appropriate clinical microbiological laboratories are available. This was added to the conclusions section (lines 621-625). We would prefer to keep this data in the manuscript, if the reviewer feels strongly that this should be moved to the supplemental data we would not object.

Conclusions

Reviewer #1: Please see comments below.

Reviewer #2: There are some other LFI tests that have been clinically used during the past outbreaks (i.e., OraQuick Ebola, SD Q Line Ebola Zaire Ag, QuikNavi-Ebola, etc.). These should be stated somewhere in the text. The authors should also explain what is the advantage of their LFI assay, compared to the previously developed immunochromatography tests.

Response: This is a good point. We added text on lines 603-619 that describes three tests that have been EUA approved and also a de novo approved RDT. We compared the TCID50/ml LoD values with our current prototype.

Reviewer #3: Due to previously reported LFI, which can broadly detect ebolaviruses, authors in this manuscript should demonstrate which points of LFI in this study could be superior to previous one: cross-reactivity and stability could be important aspects to compare.

We expanded the information on pan-reactive RDTs in lines 603-619 as also requested by review #2 above.

Minor comments:

Reviewer #1: Please see comments below.

Reviewer #2: Lines 25, 67, 69, 75, and 83: This outbreak is not called "pandemic".

"Pandemic" has been changed to "outbreak" throughout the text.

Line 423, 2HK1 (capture):2HK12 (detection): Should this be 2HK12 (capture):2HK1 (detection)?

Response: Corrected

Reviewer #3: 1. Line 344: "if "high background" was observed…. Please clarify the threshold of "high background".

Response: This was a simple visual observation of a test line upon testing control NHS. We added "visually" to line ~374.

2. Table 3: Please provide footnote explaining the value of LOD: e.g., mean or SD or SE.

Response: Added SD to the Table title.

3. Line 423: Data indicated 2HK1 (detection) and 2HK12 (capture) was optimal. Please explain why the opposite combination was selected.

Response: This was an error on our part, we switched this in the text.

Overall Comments

Reviewer #1: This paper reported a point-of-care diagnostic test for EBOV with lateral flow immunoassay (LFI) using anti-GP and VP40 monoclonal antibodies newly developed in this study. The LFI is more sensitive than commercially available LFI test kit. The LFI is highly required in the scene of ebolavirus disease outbreak. Therefore improved LFI should be shared by the medical and research community through this journal. However, I concerned several points in this manuscript for publication. I hope my comments will improve the quality of their manuscript.

Major comments:

1. In line 108-109, The authors mentioned that VP40 is most abundantly expressed viral protein. And the results of SPR data in Table 4 showed three anti-VP40 mAbs had higher affinities to the target protein than anti-GP mAbs. However, their assay could detect GP in blood samples more sensitively. I think the authors should add more discussion to explain the reason why LFI using anti-GP mAbs worked better than that using anti-VP40 mAbs. Are there any previous data which show similar results? or did sGP in the infected samples detected in this assay and increased the sensitivity of this assay?

Response: This is a interesting question. We did add to the text in lines 568-575 that VP40 may not be a form that promotes efficient binding of mAbs, as it is know to form dimers and oligomers. Also, we did mention the possibility that the GP prototype could be binding to sGP.

2. Fig1. Anti-GP mAbs detected multiple or smear bands in western blotting in Figure 1A. The authors should indicate which GP proteins or its derivative (e.g. GP1,2, GP1 or GP2) were detected aside of the figure.

Response: The recombinant protein purchased from IBT is “His-tagged recombinant EBOV GP minus the transmembrane domain (rGP) (IBT Bioservices, Rockville, MD)”. After calling IBT, technical support stated this was a full-length GP (minus TMD) recombinant protein. After an extensive literature search it appears that uncleaved GP and GP1 would be seen at the higher molecular weight band (~110 kD region of the Western blot). The lower molecular weight band on the Western (~50kD) we were told by technical support was GP1. However, this does not make sense to our team and it would appear that this

is the molecular weight of secreted GP (sGP). We would like to leave the Figure as is, so as not to not improperly label the figure. However, we would gladly take suggestions from reviewers on how to proceed.

3. Fig3. The author suggested that the samples of NHP#2 in day 7.5 resulted in positive in the result of VP40-specific LFI Prototype. However, I could not find a difference of the band intensity between sample of day 7.5 and that of day 4, which they concluded as negative.

Response: There does appear to be a faint band in VP40 specific LFI day 4 nhp #2. However, this was visually scored as negative by the reader at the time of testing and we think it is best to keep it as a negative.

4. Table 2 SD values for Background and Signal intensity determined with indicated mAbs are not shown in this Table. How many replicated experiments did you try for this result? The authors should add the information, and if necessary, they also add SD for each value.

Response: This is a good point. Only one replicate is performed for this analysis as we prefer to read the LFI in the Qiagen ESE reader at a specific timepoint as signal can vary over a small amount of time if read at multiple timepoints. In addition, this is merely a way for us to down-select the best mAb pairs to move forward for further development. We updated the text at lines 378-379 to describe this experiment.

5. Line 548, The authors hypothesized that the GP-specific LFI many be capable of detecting even lower levels of EBOV, however, there are a lot of reports which determined the viral load in the serum or blood of EVD cases. I suppose they can clearly discuss how effectively the LFI can determine the EVD cases with the clinical samples, and limitation of this assay.

Response: We added to the text on lines ~603-619. Studies have indicated that roughly $5x10^{1}$-$7x10^{7}$ RNA copies per ml of serum is present on the first few days following initial symptoms. Our goal would be to identify more patients that have lower viremia compared to some of the other tests currently used.

6. S1 Fig. Although the authors used same mAbs for GP and VP40 in Figure 1 and Figure S1, the band size of Figure S1 is not

consistent to that of Figure 1. Which size of the band are correct? VP40 is detected around 40kDa, however, the major band appeared at around 80kDa in Figure S1. It looks strange for me.

Response: Good point, this should have been explained. In Figure 1 recombinant VP40 was probed by Western blot while in Supplemental Figure S1 purified virus like particles were used. We did find the difference in molecular weights interesting, however it is well established that VP40 can form dimers and oligomers. This was explained in the text and references were added following the text that described Supplemental Figure 1 (lines 324-328).

7. Did the authors examined cross-reactivities of the mAbs for GP or VP40, at least the highly reactive mAbs used in this LFI with other ebolavirus species, e.g. Sudan, Bundibugyo EBOV or other microbes which cause acute febrile illness in the sub-Sahara Africa which is endemic region of EVD, e.g. Plasmodium, Dengue, and Typhi?

Response: Please see above. We did add Western blot data for the GP and VP40 mAbs and LFI data from the GP prototype binding to a number of ebolavirus species. Unfortunately, as mentioned above we did not perform testing on other microbes endemic to sub-Sahara Africa. This work is planned with a large panel of microbes that are in-house at InBios International.

Minor comments:

1. Line 67,69, 83; In general, the word of “pandemic” is not commonly used to represent the outbreak of Ebola in West Africa. I recommend replacing into “Epidemic” as reference 9.

Response: Corrected

2. Line 405, 100X --> 100-fold

Response: Corrected

3. Line 423, 2HK12(capture):2HK1(detection) --> 2HK12(capture):2HK1(detection)

Response: Corrected

Reviewer #3: The manuscript entitled “Development of an antigen

detection assay for early point-of-care diagnosis of Zaire ebolavirus" by DeMers HL et al. described that the lateral flow immunoassay (LFI) made with a combination of two original monoclonal antibodies against Zaire ebolavirus (EBOV) can detect EBOV antigens in culture supernatants or serum samples of infected nonhuman primates. The point-of-care tool for the detection of EBOV was previously developed in response to the need during EVD outbreak in West Africa. The LFI made by USAMRIID team could detect GP of Zaire, Sudan, Tai Forest, and Reston virus (range: 5x10^4 to 5x10^5 PFU/ml: Phan JC et al. JID, 2016, 214: S222-S228). The novel LFI described in this study could detect Zaire ebolavirus GP (LOD = 1.2 x 10^4 TCID50/ml) or VP40 (9.8 x 10^4 TCID50/ml). Although overall concept is similar to the previous study, this study reports a novel LFI system to detect EBOV VP40 or GP. The manuscript is overall well written, while some more information will likely improve the content.
